# Headache, migraine, brain lesion and MRI study

**DOI:** 10.1186/1129-2377-14-S1-P102

**Published:** 2013-02-21

**Authors:** N Radojkovic Gligic, R Amanovic Curuvija, K Kacar

**Affiliations:** 1 Hospital for cerebrovascular desease St. Sava, Serbia and Montenegro

## Introduction

Many people have primary and often disabling headaches. The common forms are migraine and tension headache.Headache in general and migraine in particular have been increased risk if comorbidities. The migraine with aura is a marker for increased risk of cerebrovascular disease, specifically stroke. Migraine has been associated with a variety of structural brain lesion, including silent infarct, like lesion in the postertior circulation territory and with white matter hyperintensities.

## Methods

Of the 120 participants with brain scans, we compared the means of continuous characteristics and frequency of categorical characteristics of participants according to the headache status. We run age adjusted multivariable models for total and localised white matter hyper intensities. The multivariable models controlled for age (continuous) sex, history of hypertension, smoking(ever, never) body mass index, alcohol consumption, serum total cholesterol level and family history of severe headache.

## Results

Characteristics of lesions were visualized simultaneusly in axial, coronal, and sagital planes. A brain infarct was defined as focal lesion of 3mm or more with the same signal characteristical as cerebrospinal fluid on both T1 and T2 weighted sequences, and these were discriminated from dilated vascular space ( Virchow-Robin space) according to their shapes and locations. We applied this definition to all lesion irrespective of location. We distinguished the infarcts in the cerebellum, brain stem and in other locations.

## Discussion

In this population we found that any lifetime history of severe headaches was associated with an increased risk of higher volumes of total, deep, and periventricular white matter hyperintensity.-for migraine and non migraine headache. Participants who had migraine with aura in general was associated with brain infarcts.
